# Lung ultrasound—a primary survey of the acutely dyspneic patient

**DOI:** 10.1186/s40560-016-0180-1

**Published:** 2016-08-31

**Authors:** Francis Chun Yue Lee

**Affiliations:** 1 Acute and Emergency Care Centre, Khoo Teck Puat Hospital, Singapore, Singapore; 2 Yong Loo Lin School of Medicine, National University of Singapore, Singapore, Singapore

**Keywords:** Lung ultrasound, A-lines, B-lines, Z-lines, I-lines, Curtain sign

## Abstract

There has been an explosion of knowledge and application of clinical lung ultrasound (LUS) in the last decade. LUS has important applications in the ambulatory, emergency, and critical care settings and its deployability for immediate bedside assessment allows many acute lung conditions to be diagnosed and early interventional decisions made in a matter of minutes. This review detailed the scientific basis of LUS, the examination techniques, and summarises the current applications in several acute lung conditions. It is to be hoped that clinicians, after reviewing the evidence within this article, would see LUS as an important first-line modality in the primary evaluation of an acutely dyspneic patient.

## Background

Lung ultrasound (LUS) is an effective and sensitive tool compared to the traditional chest auscultation and chest X-rays [1–3]. Its use as a primary survey tool in the acutely dyspneic or hypoxemic patient gives an immediate understanding of the state of the lung and influences therapeutic decisions. Proper LUS practise requires the following: the understanding of pathophysiology of acute lung conditions; the sonographic features they produce; and the ability to elucidate the LUS signs in the clinical context of the patient.

### Lung ultrasound examination

LUS examination is best performed with a low frequency transducer (3–5 MHz), such as the commonly available curvilinear transducer, set to a study depth of about 12–18 cm (depending on body habitus). Microconvex transducers have the additional advantage of a smaller footprint for better intercostal imaging and application in younger patients. High-frequency transducers are helpful for the search of lung comets and detailed visualisation of pleural layers and small subpleural lesions. The phased-array transducer for echocardiographic applications could be used, but defining near field pathology such as consolidation or atelectasis would be a challenge.

Filters, such as compounding and harmonic imaging, cancel off artifacts and noise; is unhelpful in LUS; and should be turned off. The rest of this article will introduce LUS signs that are identifiable with the first two types of transducers mentioned above.

The transducer should be applied onto the chest wall in the longitudinal cranial-caudal plane, straddling across the intercostal space, with the marker oriented towards the head. All the images; with the exception of image B in Fig. 2, which is a transverse study; presented in this article are studies in the cranial-caudal axis with the left side of image oriented towards the head.

LUS examination is performed with the patient in the supine or reclined position, starting with right anterior chest, followed by the right lateral chest, and ends with a careful examination of the lower lung and the costophrenic recesses (in this article, the term “lung base” will be used to denote these two areas); this is repeated on the left side. The posterior lung should also be examined with the patient turned or in the sitting position. During LUS examination, the transducer should be held still for a few seconds observation, avoiding unnecessary movements. Care must be taken to keep the probe perpendicular to the chest wall during scanning. Excessive tilting or angulation may orientate the ultrasound beam out of the plane of the lung, producing uninterpretable images; this is especially a problem at the clavipectoral triangle and axillary area.

**Fig. 1:**
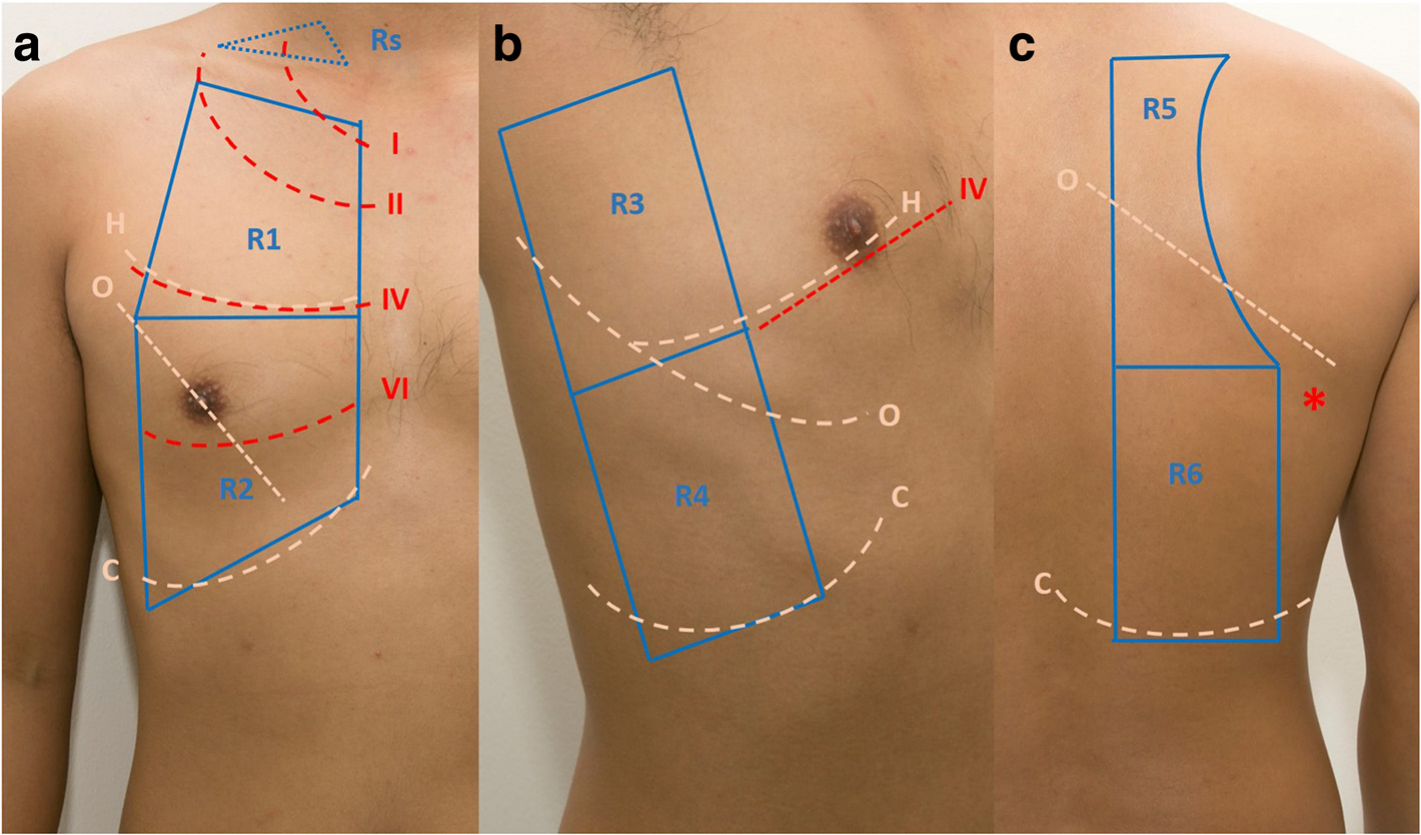
Scanning sectors (as used at the author’s centre). Zones on the right hemithorax. **a** *R1* right anterior upper zone, *R2* right anterior lower zone, *Rs* right supraclavicular fossa **b** *R3* right lateral axilla zone, *R4* right lateral lower zone **c** *R5* right posterior upper zone *R6* right posterior lower zone. *I*, *II*, *III*, *IV* first, second, third, fourth ribs, respectively, *H* horizontal fissure, *O* oblique fissure, *C* costophrenic recess, lowest limit of LUS study where curtain sign is found, *inferior angle of scapula

There are several approaches to studying, documenting and communicating LUS findings. At the author’s centre, each hemithorax is divided into six sectors for study (Fig. 1 and Table 1). Other methods, dividing the chest into sectors or quadrants [4, 5]; using the anatomical lines [6] as a guide; and marking three key scanning points on the chest [7] have been proposed.

**Table 1:** Detailed descriptions of scanning sectors in LUS

Chest	Sector	Boundaries	Anatomical/study significance
Anterior	R1 or LI (anterior upper)	Upper: clavicle; lower: 4th rib; medial: sternal edge; lateral: defined by LUS image of lung; beyond this border are contents of the axilla and clavipectoral triangle	• Horizontal fissure is in line with the 4th rib; therefore, this zone contains the upper lobe of the lung
	R2 or L2 (anterior lower)	Upper: 4th rib; lower: variable, depending on body habitus and defined by curtain sign in LUS and appearance of abdominal contents;liver on the right side, bowel and spleen on the left; medial: sternal edge; lateral: anterior axillary line	• The 6th rib approximates the inferior most part of the lung and anterior insertion of diaphragm (not seen in normal LUS with curtain sign). Beyond the 6th rib is the potential space: costophrenic recess • This sector contains mainly the middle lobe; on the left lung, lingular lobe; with a small portion of the lower lobe on the lateral side • The sector on the left hemithorax is very small due to the position of the heart; no sector in cardiomegaly states
	Rs or Ls supraclavicular fossa	Triangle formed by the clavicle, lower parts of sternomastoid and trapezius	• Optional study area for the following: first rib; apical pneumothorax; pulmonary tuberculosis
Lateral	R3 or L3 lateral axilla	Upper: axilla; lower: the axis of the 4th rib; anterior: anterior axillary line; posterior: posterior axillary line	• This sector contains primarily the upper lobe of the lung with a small portion of the lower lobe
	R4 or L4 lateral lower	Upper: the axis of the 4th rib; lower: variable, depending on body habitus. Defined by curtain sign in LUS; anterior: anterior axillary line; posterior: posterior axillary line	• This sector contains primarily the lower lobe of the lung
Posterior	R5 or L5 posterior upper	Upper: defined by LUS image of the lung; medial: thoracic spine; lateral: medial border of scapula; lower: level of the inferior angle of the scapula	• This sector contains the upper lobe and lower lobe of the lung in almost equal proportion
	R6 or L6 posterior lower	Upper: level of the inferior angle of the scapula; medial: thoracic spine Lateral: posterior axillary line; lower: defined by curtain sign in LUS and appearance of abdominal contents ; liver on the right side; bowel and spleen on the left	• This sector contains primarily the lower lobe of the lung

Regardless of the study convention used, thorough scanning is important. This must include the posterior lung and the lung bases, as acute disease processes commonly start in these areas.

### Basic lung signs

#### Pleural line—the starting point

**Fig. 2:**
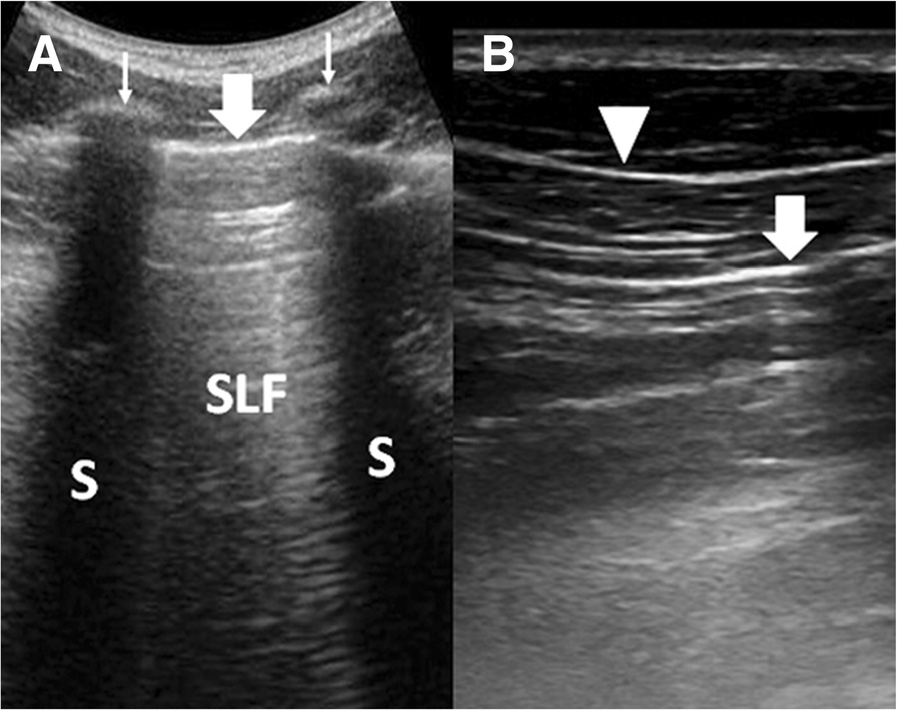
Comparing two scanning planes in LUS. **a** LUS performed in the longitudinal or cranio-caudal plane showed ribs (*thin arrows*) and their acoustic shadows (*S*). Just below the level of the ribs is the pleural line (*thick arrow*) and the sonographic lung field (SLF). **b** Subcutaneous tissue lines (*arrowhead*) could be mistaken for the pleural line (*arrow*) when LUS is performed in a transverse plane, without the guidance of the rib structure

Identification of the pleural line is the first step in LUS. It is important to start with the transducer positioned in a longitudinal plane (cranio-caudal axis), straddled across the intercostal space and the ribs. The ribs will serve as a guide to the correct identification of the pleural line and avoid confusion with hyperechoic lines cast by tissue planes (Fig. 2); and for this reason, the transverse oriented study is not recommended as a start. Without visualising the pleural line, one cannot be certain that the lung is being examined. The area below the pleural line and between the acoustic shadows cast by the ribs is the sonographic lung field (SLF), the focus of LUS examination.

#### Lung sliding

The first question in LUS evaluation is whether there is lung sliding. In the normal lung, where the visceral and parietal pleural are closely applied and slides with respiration, an artifact known as lung sliding is generated. This appears as an actual movement, shimmering or flickering at the pleural line, depending on how the transducer beam interacts with the pleural line. Lung sliding may be subtle: at the end of the respiratory cycle; near the apex of the lung which has much less respiratory excursion; in clinical situations of hypopnea and bradypnea. The subject should be asked to take deliberate breaths to overcome this problem; otherwise, the operator should patiently observe the pleural line. The key to lung sliding observation is to train the eyes on the pleural line and not be distracted by other disturbances such as chest wall movements.

#### The appearance of lung air on ultrasound

**Fig. 3:**
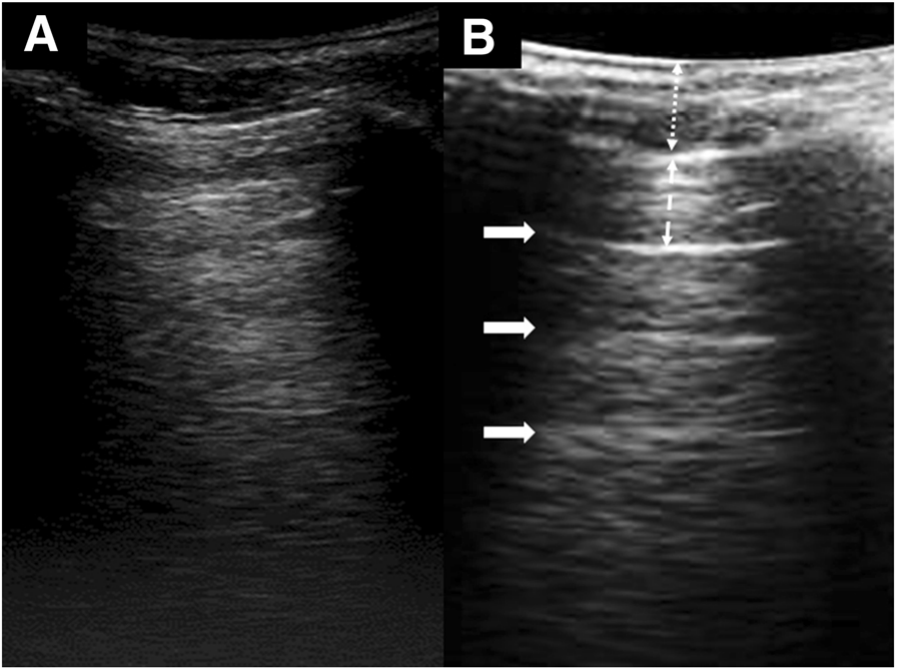
Two different appearances of air in LUS. **a** A hyperehoic appearance of lung air without A-lines. **b** LUS appearance with A-lines (*solid arrows*). The distance between the A-lines (*dashed arrow*) is equal to that between the transducer and the pleural line (*dotted arrow*). A-lines, other than that indicating a strong reflector is present, have no clinical significance

Air has low acoustic impedance to ultrasound. When ultrasound traverses from tissue to air, the large acoustic impedance mismatch causes 99 % of the ultrasound to be reflected, resulting in a hyperechoic image in the SLF (Fig. 3). In the backdrop of this image, a number of LUS artifacts have been described and classified using an alphabetical system [8, 9]. Four basic artifacts of fundamental importance to LUS practise, with distinct mechanism of genesis, are described below with their corresponding alphabetic nomenclature.

##### A-lines (repetition artifacts)

When the incident ultrasound wave is perfectly perpendicular to a highly reflective surface, it will be reflected back and forth between the transducer face and the reflector (c.f. short-paths reverberations), creating repetition artifacts consisting of a series of equally spaced horizontal lines [10, 11]. The distance between each horizontal line is equal to that between the transducer and the reflector, but the strength of the images decrease with depth as energy is lost through repetitive reflections. Examples where repetition artifacts could be generated include:

- Inner layer of the trachea in airway scanning
- Needle in ultrasound guided procedures
- Trapped air bubble within a condom in transvaginal or endorectal scanning [10]

The pleural line is a strong reflector creating similar repetition artifacts. A-lines is a specific name given to these artifacts found in LUS. The significance of A-lines is simply that the ultrasound has encountered a strong reflector and in itself has no clinical meaning.

##### Lung comets (I-lines)

**Fig. 4:**
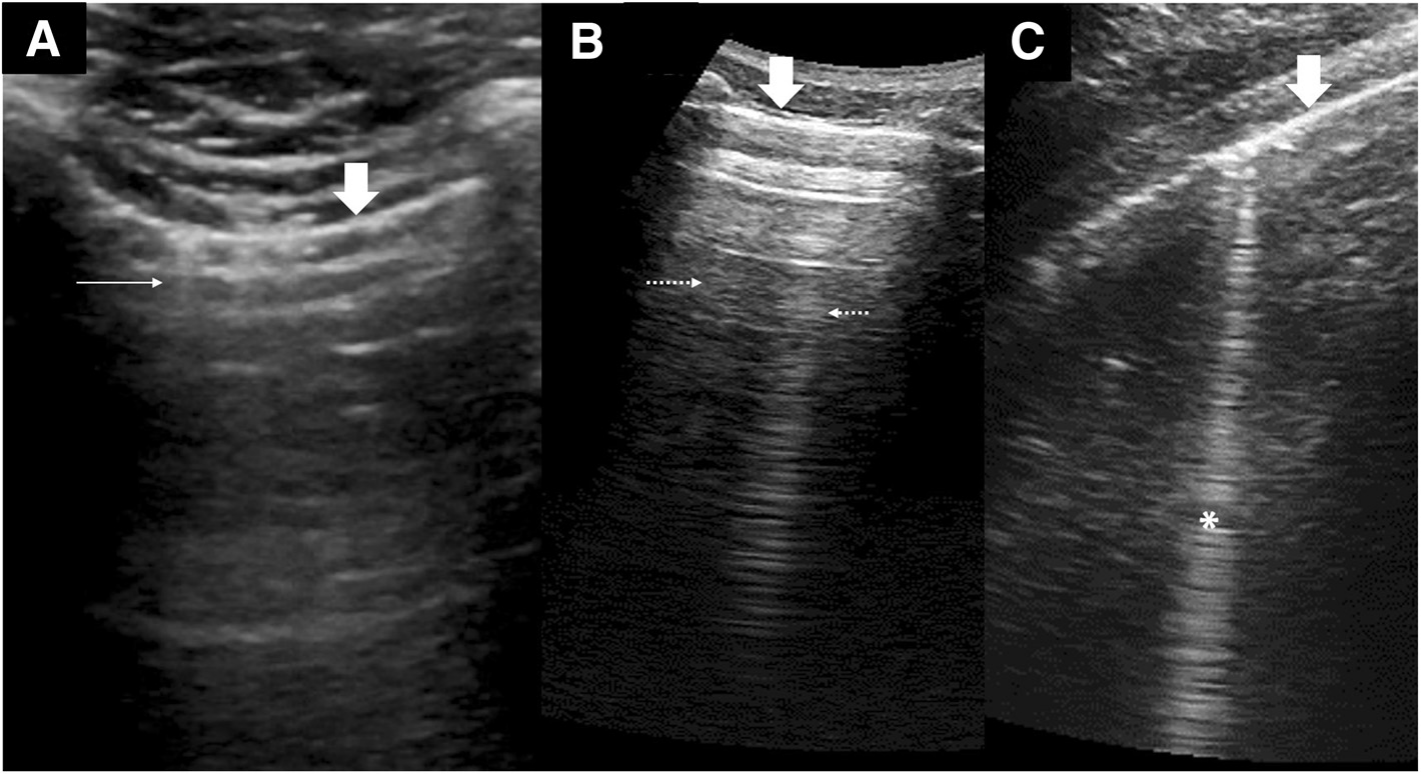
Vertical artifacts in LUS. **a** Lung comet (*thin arrows*) or I-lines arising from the pleural line (*thick arrows*) as seen with a high-frequency transducer at 8.5 MHz. **b** Static reverberation artifacts or Z-lines (*dotted arrows*) within the SLF are weak images with no relationship with the pleural line (*thick arrow*) and fades with depth. **c** A strong ring-down artifact or B-line (*asterisk*) starts from the pleural line (*thick arrow*) and reaches the depths without fading. It also swings side to side with lung sliding

In the right conditions, when ultrasound waves are trapped within small confines of tissue or structure, short-paths reverberations [10] could occur, producing short vertical artifacts that fades with increasing depth. These are the characteristics of the comet-tail artifact [12, 13] (Fig. 4). In LUS, the important type of comet-tail artifacts are the ones that start from the pleural line and move with lung sliding and this property indicates their likely origin from peripheral lung intersititia. They share similarity to B-lines (described below), albeit being short in length and weak in appearance. They are commonly referred to as lung comets or simply comet-tail artifacts (an unrefined term). The closest description of this artifact in Lichtenstein’s alphabetical nomenclature is the I-lines [7, 8]. An important point to note is that lung comets (I-lines) are readily seen with high-frequency linear transducer but difficult to visualise with low frequency transducers. The presence of lung comets is a good confirmation that the two pleural layers are in contact, which is useful in excluding a pneumothorax. However, because lung comets are commonly seen in normal lung, they cannot be used in lung intersitial disease diagnosis.

##### Z-lines (reverberation artifacts)

These artifacts could be randomly found in any part of the lungs during LUS exam and are likely to be caused by short-paths reverberations between the parietal pleural and the endothoracic fascia. Because of the extra-pulmonary location, they are often seen as static vertical artifacts which do not move with lung sliding. Z-lines do not have any clinical significance except that they could easily be misinterpreted as B-lines (Fig. 4).

##### B-lines (ring-down artifacts)

The ring-down artifact (Fig. 4) has been shown to be generated by a bubble-tetrahedron bugle mechanism [11, 14]. When a series of bubble-tetrahedron (small amount of fluid is trapped between four microbubbles) are aligned, they form a “bugle” that is able to oscillate continuously when struck by ultrasound, persistently emitting signals back to the transducer. The resulting effect is a strong vertical artifact consisting of closely spaced horizontal echoes that “ring-down” the end of the screen.

The ring-down artifact within the lungs could only arise from the subpleural interstitia, intralobular interstitia, interlobular septa, and interlobar fissures where the conditions for forming bubble-tetrahedral complexes are potentially available.

In most part of the normal lung, air predominates and the parenchyma does not provide sufficient acoustic windows to generate ring-down artifacts. In contrast, the lung bases [15] where hydrostatic pressure gives a more fluid-rich interstitia; at fissures where there is abundance of connective tissue with pulmonary vasculature, ring-down artifacts could be seen. These normal ring-down artifacts are usually thin and transient (changes with posture), and there should not be more than three within one SLF or intercostal space.

Disease processes that alter the fluid-air composition of the interstitia and alveoli provide the environment to form bubble-tetrahedron bugles and, hence, generate ring-down artifacts. Ring-down artifacts arising from the diaphragmatic pleural have also been shown to be useful in the diagnosis of pulmonary diseases [16], a sign of interstitial involvement in the lung bases as examined through the liver window. B-lines is a term used to describe ring-down artifacts found within the SLF in LUS. B-lines are technically not comet-tail artifacts contrary to popular nomenclature.

##### Basic LUS artifacts: a comparative summary

Terms like comet-tail artifacts, lung comets, and B-lines are often used loosely and interchangeably in current LUS literature, creating some confusion and the misunderstanding. Taking reference from the mechanism of artifact generation, a comparative summary is made to help readers understand the nuances (Table 2).

**Table 2:** Basic LUS artifacts: a comparative summary

	A-lines	Lung comets (l-lines)	Z-lines	B-lines
Artifact generation mechanism	Repetition: Long-paths reflections between transducer and reflector.	Reverberation: Short-paths reflections within tissue structures or materials.		Ring-down: Bubble-tetrahedron mechanism.
Characteristics	Short, repetitive equidistance horizontal lines. Fades with increasing depth.	Weak vertical artifacts comprising of irregularly spaced horizontal lines. Artifacts are often of variable length and fades with increasing depth. This is the typical description of a comet-tail artifact.		Strong narrow vertical artifact comprising of tightly spaced short horizontal lines. Artifact starts from point of origin to the end of the ultrasound screen. Does not fade with increasing depth. This is the typical description of a ring-down artifact.
Artifact generation mechanism in LUS	Ultrasound encounters the pleural line (strong reflector).	Created in the lung interstitial or in the? Interpleural layer.	Originate from an extra-pulmonary location probably between the parietal pleural and the endothoracic fascia.	Created by “thickened” lung interstitia and interlobular structures near lung surface parenchyma.
Additional characteristics in LUS	Found within SLF.	Weak vertical artifact readily found within SLF with high-frequency transducers. Similarity to B-lines: • l-lines arise from the pleural line. • Move with lung sliding/respiration. Unlike B-lines: • Short, often <2 cm.	Found within SLF • Does not appear to be related to the pleural line. • Static: does not move with lung sliding or respiration. • Blend with other background artifacts.	Found within SLF. **•** B-lines arise from the pleural line. **•** Move with lung sliding/respiration, like search lights. **•** Dominant over other \background artifacts (e.g., A-lines, Z-lines).
Significance	Signifies ultrasound interaction with a highly reflective surface. No diagnostic significance.	Signifies that the pleural layers are in contact. Important in pneumothorax diagnosis. As they are commonly found in the normal lung. Cannot be used for diagnosis of lung interstitial diseases.	No clinical significance. The uninitiated may mistake these for B-lines.	When more than 3 per SLF, may signify an interstitial disease process. Important in LUS diagnosis.
Mimics in LUS	Linear foreign body in subcutaneous area.	• Pockets of air in the subcutaneous tissue may give rise to reverberation artifacts, crossing the pleura lines and into the SLF. These are also called E-Lines or “emphysema lines” [8]. • Foreign body in subcutaneous area.	• Subcutaneous emphysema could generate this artifact, originating from the subcutaneous area, instead of the SLF. • Linear foreign body in subcutaneous area.	Pockets of air in the subcutaneous tissue may give rise to ring-down artifacts, crossing the pleura lines and into the SLF. These are also called E-lines in Lichtenstein's publications [7, 8].
Other common names (better terms in italics)	Reverberation artifact.	Comet-tails, *lung comets*	*Comet-tails*, lung comets	Comet-tails, lung comets

As could be seen from this matrix, many lung conditions share similar LUS signs. It is the knowledge of extent, combination, and distribution of the lung signs that will help achieve a more accurate diagnosis. Legends: bilateral—seen in both left and right lung; symmetrical—distribution pattern in left and right lungs are similar; patchy—uneven distribution within one lung where one part of the lung is involved while others spared; focal—localised to one area of the lung, one lobe or one lung; normal (for pleural line)—thin and smooth appearance; uneven—pleural line of varying thickness

#### Curtain sign at the lung bases—the end point

**Fig. 5:**
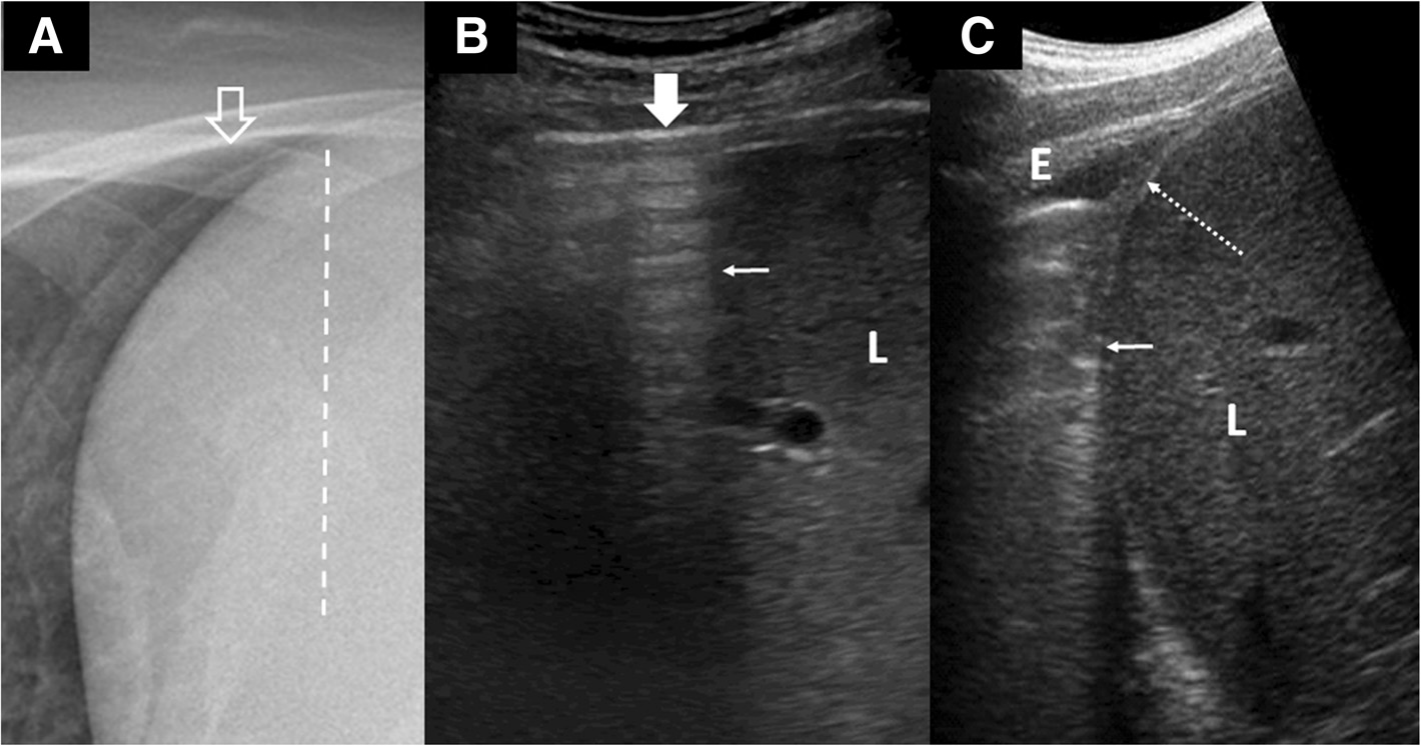
Curtain sign. **a** Chest X-ray illustrates the extent (*dotted line*) to which the lower parts of the lung (*open arrow*) cover the abdomen. **b** LUS shows the pleural line (*solid arrow*) ends abruptly with an edge (*thin arrow*) forming an acoustic shadow,  the “curtain sign,” which slides over the liver (L) with respiration. The lateral diaphragm is always hidden by the curtain and not seen in normal LUS. **c** An example of an abnormal curtain sign: a small effusion (*E*) causing an incomplete “curtain” sign (*thin arrow*) and exposing the lateral diaphragm (*dotted arrow*)

As one survey from the upper lung to the lung bases, one could appreciate that the SLF ends abruptly with a sharply demarcated edge called the curtain sign (Fig. 5). A normal curtain sign must have two characteristics. First, it must be dynamic, i.e., moves to and fro with respiration. Second, the unique anatomy of the thorax (and hence the lung) overlapping the abdomen means that the lateral diaphragm is always hidden under the curtain. Any deviation from these characteristics causes an abnormal curtain sign and should alert one that a change in the lung or pleural anatomy at the lung base has occurred. A careful review of that area is necessary to define the actual pathology.

#### A normal lung ultrasound study

A normal LUS study is therefore defined as

- The presence of lung sliding
- Demonstration of the typical appearance of air in the SLF of the entire lung
- The presence of the normal curtain sign at the lung bases

### Lung ultrasound signs of disease

**Fig. 6:**
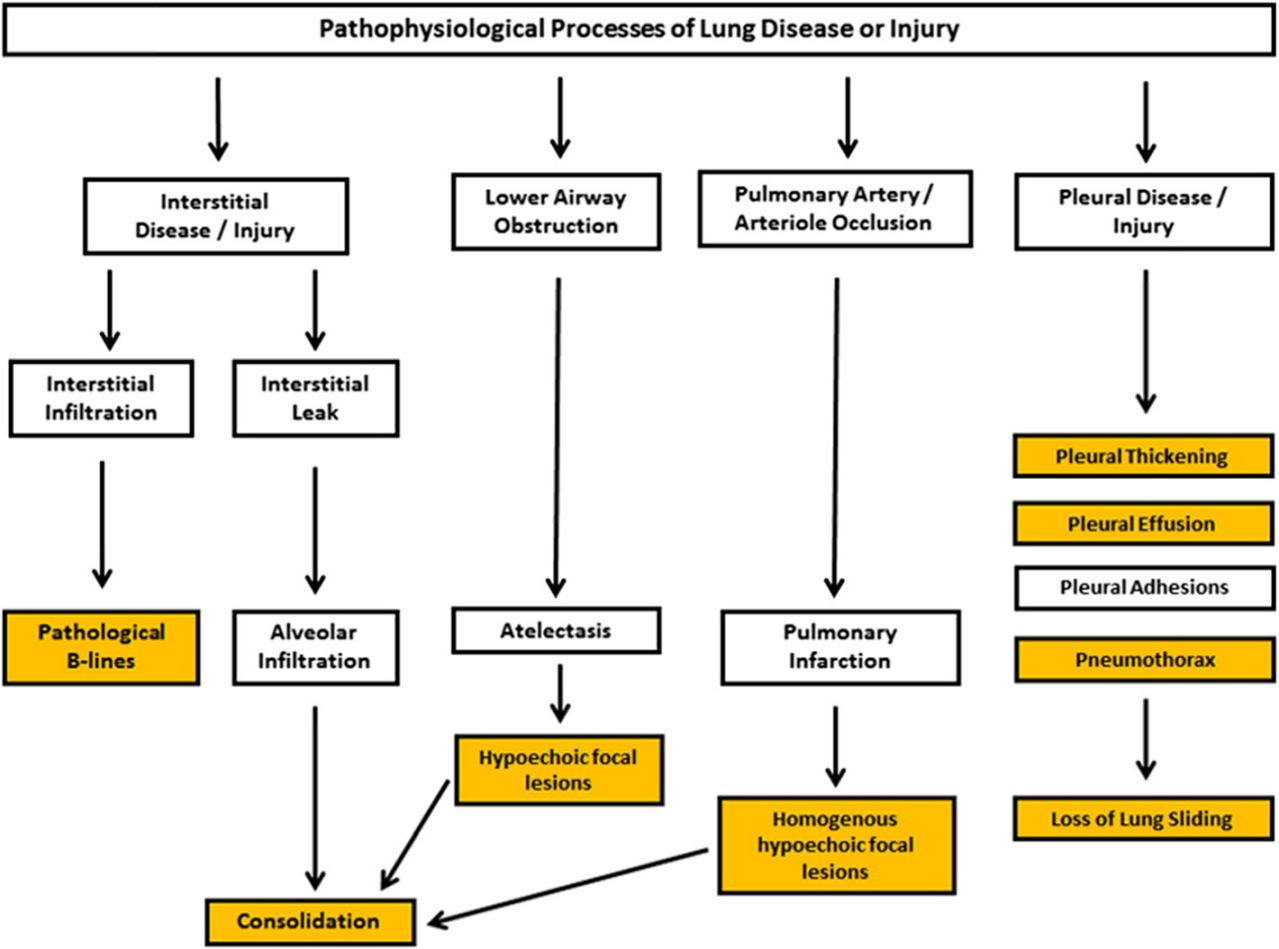
Pathological processes of lung disease and injury. This summarises some of the common endpoints of the pathological processes of lung disease and injury. The endpoints result in discernible features (*yellow boxes*) in LUS

The lung can be diseased or injured in many ways but all share some common pathophysiological end points (Fig. 6). Many of these endpoints involve the alteration of air contents within the lung tissues leading to the loss of sonographic picture of air, creates new artifacts, and opens up acoustic windows for ultrasound access.

#### Loss of lung sliding

A loss of lung sliding occurs when there is no dynamic interaction of the parietal and visceral pleural. The possible causes are summarised in Table 3.

**Table 3:** Causes of loss of lung sliding

Pleural separation	Pleural adhesions	Non-ventilation
Pneumothorax Pleural effusion Pleural diseases *Artifact mimics* * Subcutaneous emphysema* * Tissue planes*	Inflammatory adhesions • Pneumonia • Acute lung injury Pleurodesis Pleural fibrosis • Interstitial lung disease • Fibrotic lung disease Pleural diseases	Apnea Severe hyperinflation • Asthma • Chronic obstructive pulmonary disease (COPD) Non-ventilated patient • cEndotracheal tube complications • One-lung intubation Atelectasis

**Fig. 7:**
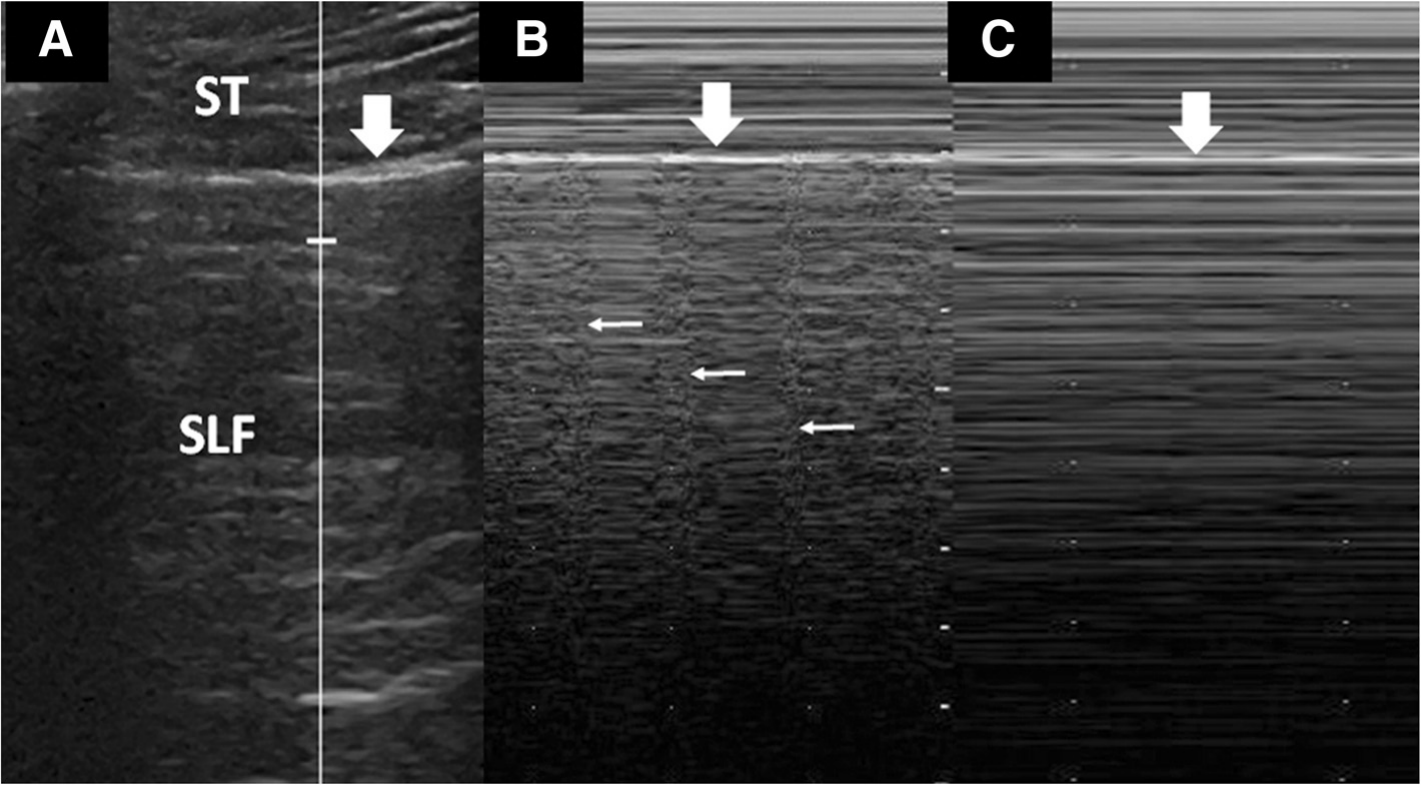
M-mode studies of lung sliding. **a** A proper M-mode study begins with the cursor (*vertical line*) centred over the SLF. The pleural line (*thick arrow*) separates the extra-pulmonary soft tissues (*ST*) and the SLF. **b** The M-mode showing “seashore” sign, where the quiet ST tracing (“sea”) is separated by the pleura line (*thick arrow*) from the noisy SLF tracing (“sandy shore”), caused by lung sliding. At regular intervals, the lung pulse (*thin arrows*) is seen. **c** M-mode showing “stratosphere” sign. The SLF tracing is “quiet” as there is no activity (lung sliding) at pleural line. There is also no lung pulse in this image

The M-mode is sometimes used to document the nature of lung sliding (Fig. 7). The M-mode image of the SLF is a grainy pattern of movement artifacts generated by lung sliding, in contrast to the extra-pulmonary soft tissues which presents a quiet and still linear image. This pattern is known as the “seashore sign” which indicate the presence of lung sliding. When lung sliding is absent, the M-mode does not register any disturbance at the pleural line and therefore gives a quiet trace of SLF. This pattern is called the “stratosphere” sign. In practise, lung sliding is best appreciated by careful visualisation of the pleural line rather than an M-mode study.

#### Conditions causing pathological B-lines

**Fig. 8:**
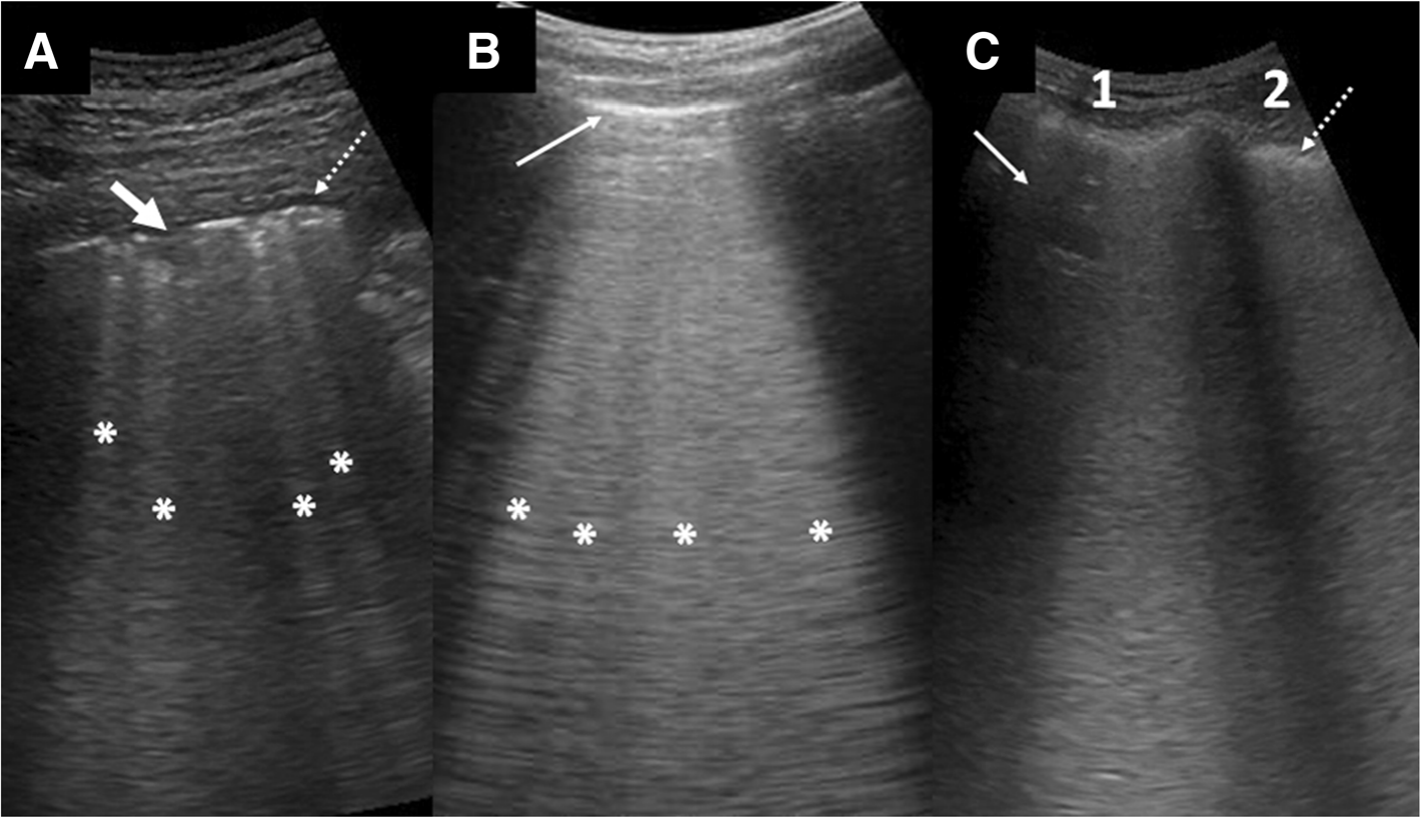
Examples of conditions with B-lines. **a** Pneumonia with several LUS features: B-lines (asterisk) of uneven spacing, a small consolidation (*arrow*), and small effusion (*dotted arrow*). **b** Cardiogenic pulmonary edema with many evenly spaced B-lines (*asterisk*) banded together into a thick sheet. Note the smooth and thin pleural line (*thin arrow*). **c** ARDS with dense B-lines involving two intercostal spaces (1, 2). Note that an area in 1 (*arrow*) is spared, indicating the patchy distribution of the disease process. The pleura is thickened and uneven (*dotted arrow*)

B-lines in lung diseases (Fig. 8) are caused by pathological thickening of the lung interstitia and septa. They are sensitive markers of interstitial lung involvement, appearing way before chest X-rays (CXR) changes but are non-specific as they could be seen in a myriad of acute and chronic lung conditions (Table 4). Acute interstitial syndrome [15] is a term used in the BLUE protocol [7, 17] to describe LUS findings of predominantly B-lines in the SLF.

**Table 4:** Conditions producing pathological B-lines

Fluid	
Cardiogenic pulmonary edema	
Fluid overload states	
Inflammatory	
Acute lung injury/pneumonitis	
Acute respiratory distress syndrome	
Pneumonia	
Fibrosis	
Pulmonary fibrosis	
Chronic interstitial lung disease	
Trauma	
Pulmonary contusion	
Blast lung	

#### Effusion

LUS was first used for the detection of pleural effusion [18] and is an important tool in the investigation of opacities in CXR [19]. Pleural effusion typically gives a hypoechoic zone in LUS with compressive atelectasis of the lung, making the visceral pleura visible (called the lung line, not pleural line). The separation of the two pleural layers also means that lung sliding is lost. The earliest LUS indication of pleural effusion is the abnormal curtain sign at the lung bases (Fig. 5). It may be possible to predict the cause of the effusion by the presence of other LUS features (Table 5), but these are non-specific signs.

**Table 5:** Other signs described in pleural effusion [8, 37, 38]

Signs	Cause	Significance
Quad sign	Pleural effusion	Shape of effusion collection
Spine sign	Large effusion	Large effusion allowing visualisation of the spine
Plankton sign	Blood, fibrin	Hemothorax, exudate
Air-fluid level	Air	Air within effusion
Jellyfish sign	Compressive atelectasis	No consolidation of underlying lung
Sinusoid sign	Jellyfish sign in M-mode	No consolidation of underlying lung
Suspended microbubble sign	Air within lung abscess	Distinguish lung abscess from empyema

LUS can ensure the safety of a thoracocentesis by defining the level of diaphragm and determining the appropriate size of effusion for safe aspiration [20]. Another important application is the investigation of the lung underlying the effusion [19].

#### Consolidation

**Fig. 9:**
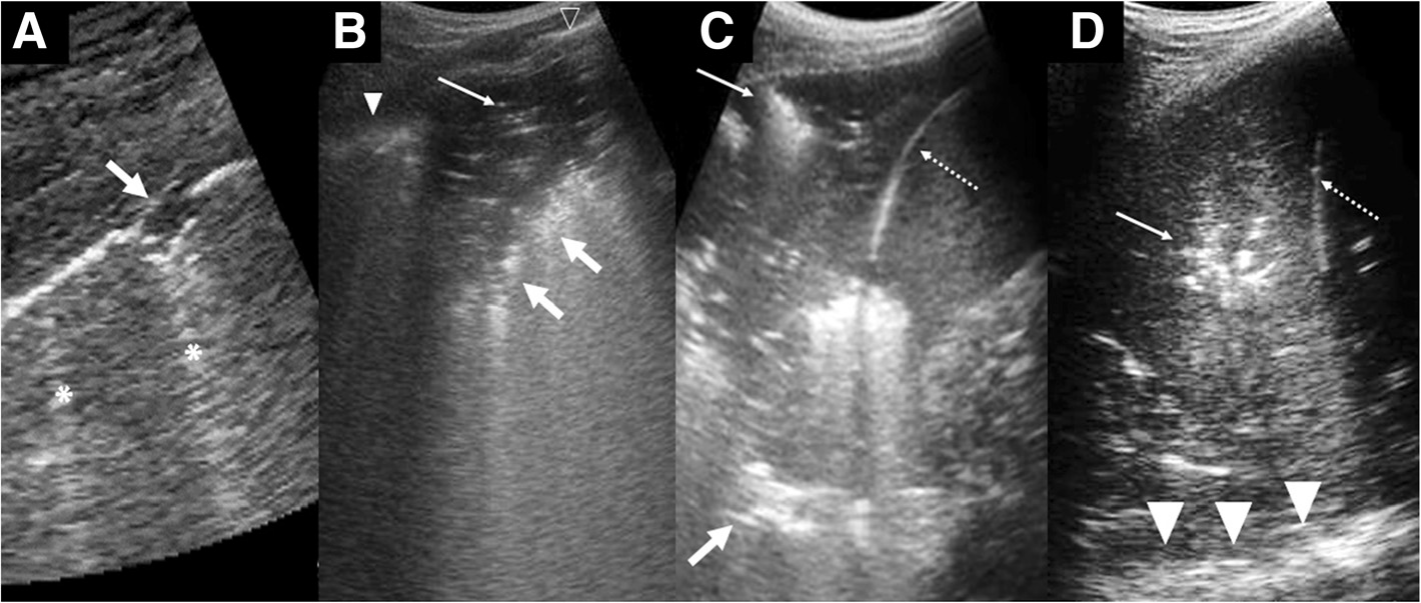
Features of consolidation. **a** Small consolidations appearing as subpleural defects (*arrow*). Ring-down artifacts or B-lines are also present (*asterisks*) **b** Wedge-shaped hypoechoic consolidations with trapped air within (*thin arrow*) and shred sign (*thick arrow*). A normal looking pleural line (*open arrow head*) and a thickened uneven pleural line (*arrowhead*) are shown. **c** A larger consolidation showing shred sign (*thick arrow*) and air bronchogram (*thin arrow*). Because this occurs at the lung base, the diaphragm (*dotted arrow*) is shown and hence the curtain sign is loss. **d** A lobar consolidation at the lung base showing air bronchogram (*thin arrow*), diaphragm (*dotted arrow*), and spine sign (*arrowhead*)

In consolidation (Fig. 9), the air in the alveoli is replaced by fluid, inflammatory exudates, and cellular infiltrates. This process removes the acoustic impedance posed by air and thus allows visualisation of the affected lung parenchyma itself. Early small consolidations appear as subpleural defects and gradually enlarge to assume a wedge-shaped appearance as more lung parenchyma is involved. The interface of the consolidation with the unaffected aerated lung creates an irregular hyperechoic border called the shred sign. A fully formed consolidation appears solid, “liver-like,” and very often with pockets of trapped air (air-alveologram) and highlighting air-filled bronchioles (air bronchogram) [21]. In consolidation, the bronchioles are often patent and communicate with the large airways. This gives rise to an air bronchogram sign that changes with respiration, called dynamic air bronchogram [22]. Hypoechoic tubes known as fluid bronchogram have also been observed. The discovery of hypoechoic regions within the consolidation may signify lung necrosis and the formation of an abscess. Consolidations involving the base of the lung, in addition, cause abnormal curtain sign.

Consolidation is a common endpoint of many disease processes such as pneumonia, atelectasis, infarction, and tumour infiltration; correlation with the clinical information of the patient is necessary to arrive at a diagnosis.

#### Atelectasis

**Fig. 10:**
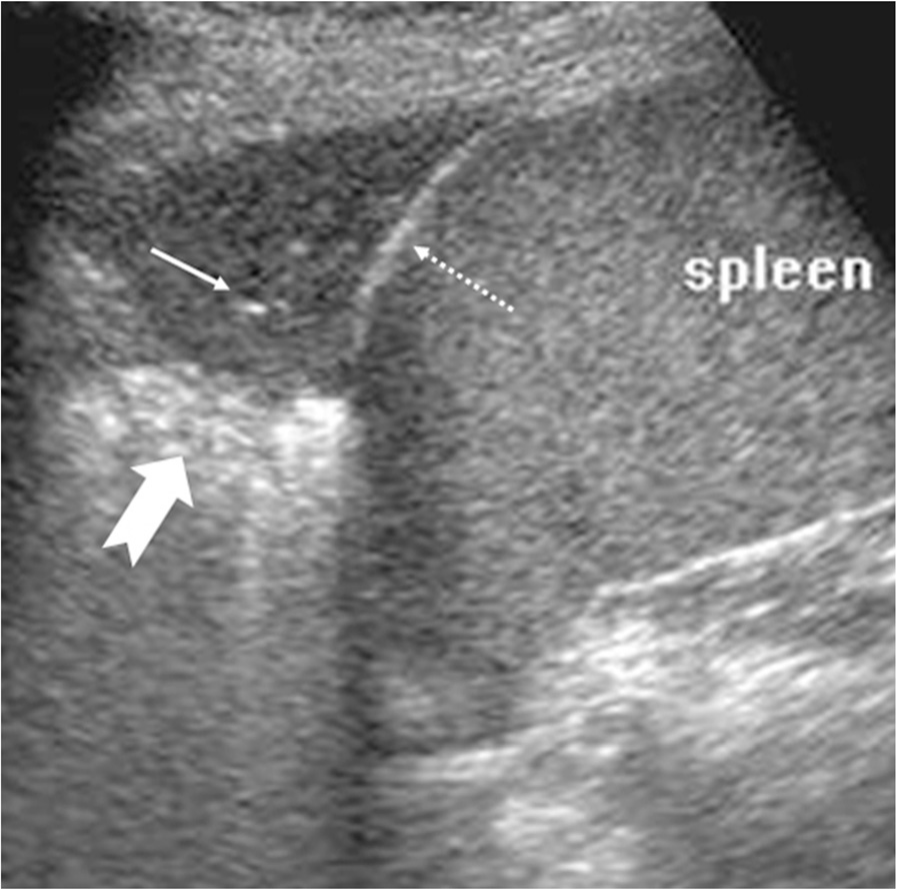
Atelectasis. Hypoechoic homogenous lesion at the lung base with air bronchogram (*thin arrow*) and shred sign (*thick arrow*). The diaphragm (*dotted arrow*) is seen as the curtain sign is lost

Atelectasis or pulmonary collapse is defined as the absent of air in parts or whole of the lung. It can be divided into compressive atelectasis caused by a large effusion and obstructive atelectasis caused by lower airway obstruction. In compressive atelectasis, the underlying lung is not consolidated and therefore will change in shape with respiration, demonstrating jellyfish sign and sinusoid sign (Table 5). LUS is an accurate tool for the diagnosis of obstructive atelectasis [23]. The early appearance of atelectasis is a homogenous liver-like lesion (Fig. 10) with loss of lung sliding. Any air trapped within the atelectasis could form a static air bronchogram. The junction between atelectasis and the aerated lung may show the shred sign. Overtime, the atelectatic segment may evolve to assume the appearance of a consolidation but the dynamic air bronchogram could help distinguish the two entities [22]. Potential use of LUS in the monitoring and management of atelectasis in the intensive care unit has been demonstrated [24].

### The lung ultrasound primary survey of the acutely dyspneic patient

A LUS primary survey refers to using the ultrasound as the initial assessment tool for a breathless patient in lieu of the traditional stethoscope or CXR. This approach requires a thorough study of the accessible lung surfaces to determine:

- Morphology (types of lung signs)
- Distribution of the lung signs

A good understanding of the pathophysiology and time course of pulmonary diseases will help the clinician anticipate the type of signs that could be expected during LUS. These signs must in turn be interpreted with the clinical findings and investigations such as lab tests, blood gases.

The lung signs matrix (Table 6) summarises the combination of LUS findings that could be found in various acute lung conditions.

**Table 6:** The lung signs matrix

Diagnosis	Lung signs distribution	Lung sliding	Pleural line	B-lines	Effusion	Consolidation
Acute pulmonary oedema/fluid overload states	Bilateral/symmetrical	Present	Normal	Bilateral/symmetrical	Often present	No
Acute respiratory distress syndrome	Bilateral/asymmetrical/patchy	Present/may be absent in severe states	Thickened/uneven	Patchy distribution	May be found	Yes
Bacterial pneumonia	Usually unilateral/focal/patchy	Present/may be absent in severe states	Thickened/uneven	Focal	May be found/empyema	Yes
Viral/atypical pneumonia	Bilateral/asymmetrical/patchy	Present/may be absent in severe states	Thickened/uneven	Patchy distribution	May be found	Yes
Acute interstitial lung disease	Bilateral/asymmetrical/patchy	Present/may be absent in severe states	Thickened/uneven	Patchy distribution	May be found	Yes
Pneumothorax	Focal/starts with upper lung	Absent	Normal	No	No	No
COPD exacerbation/asthma	–	Present	Normal	No	No	No
Acute pulmonary infarction	Focal	Present/may be absent	Normal/may be thickened	No	May be found	Yes—at late stage
Atelectasis	Focal	Absent	Normal	No	May be found	Yes—at late stage

A LUS workflow in acute lung disease diagnosis called the BLUE protocol [7, 17] could also be used. This protocol was validated in a study population with single diagnoses with confounding cases (rare; more than one diagnoses; no diagnoses) excluded, and the authors acknowledged the limitations of the protocol in separating disease entities that share similar lung signs. The aspects not covered by the BLUE protocol were explained in detail in a subsequent publication [25].

#### Pneumothorax

Pneumothorax is the signature condition with loss of lung sliding. In a dyspneic patient with a previously normal lung, the loss of lung sliding is predictably due to pneumothorax and the presence of lung sliding rules out pneumothorax with a >99 % sensitivity [26, 27].

Distinguishing pneumothorax from situations involving a relatively aerated lung (such as non-ventilation, pleurodesis) is often a challenge. One scenario that illustrates the non-specificity of loss of lung sliding; in an intubated asthmatic patient with worsening hypoxemia, the finding of loss of lung sliding in one lung could be attributed to either a pneumothorax or non-ventilation of the lung (e.g., slipped endotracheal tube). Conditions such as pleural effusion, consolidation, and atelectasis are easily distinguishable from pneumothorax because there are other ultrasound features to support the respective diagnoses.

In difficult situations, additional lung signs are needed, all with aim of determining whether the two pleural layers are in contact:

##### B-lines

Since B-lines originate from the lung interstitia, the demonstration of B-lines signifies that the lung is fully inflated and the visceral pleura being in contact with the parietal pleura. A pneumothorax, no matter how small, will obscure any existing B-lines. The pitfalls of using B-lines as evidence are:

- They are rare in healthy lungs (without parenchymal disease), especially the upper lung, where a pneumothorax usually manifests
- There are also uncommon situations where B-lines are seen with a pneumothorax: adhesions of parts of the lung in a loculated pneumothorax [28], pneumothorax in lung fibrosis and failed pleurodesis

##### Lung comets (I-lines)

As explained earlier, I-lines share the same origin and some characteristics as B-lines but are generated through a different mechanism. They are readily seen in healthy lungs (without parenchymal disease) with a high-frequency transducer and their presence means the pleural layers are in contact. The main pitfall of the search for I-lines is that they are often not visible with a low frequency transducer.

##### Lung pulse

Lung pulse (Fig. 7) is an artifact seen in M-mode studies of the lung. It is due to cardiac pulsations conducted to the chest wall, which register a disturbance to the transducer. Logically, the lung pulse is not seen when the pleural layers are separated in a pneumothorax. Two pitfalls of lung pulse as a supporting sign are:

- The lung pulse is sometimes not seen in a normal lung because of patient factors, such as body habitus
- A very small pneumothorax may still allow the transmission of vibrations through the pleural cavity air to the transducer, registering a lung pulse

##### Lung point

The lung point is the junction between the pneumothorax and the normal lung. In B-mode LUS, one part of the pleural line demonstrates lung sliding and the other does not. The lung point also represents the extent of the pneumothorax discoverable by ultrasound. In M-mode, there is an alternating seashore sign with stratosphere sign. However, this M-mode feature is also seen in patients with bradypnea where there are long intervals without respiratory movements; therefore, finding and visualising the lung point in B-mode is still a better approach.

Pitfalls of lung point are:

- A large pneumothorax involving the entire lung does not have a lung point
- It is difficult to find a lung point in a person with pneumothorax on top of a pre-exisiting condition of pleural adhesion (e.g., fibrotic lung disease, failed pleurodesis)

#### Acute respiratory distress syndrome vs acute cardiogenic pulmonary edema

Acute lung injury often goes through the stages of interstitial involvement, thus generating B-lines, to the stage of alveolar infiltration and, hence, consolidations. The severe end point is the clinical state of acute respiratory distress syndrome (ARDS). This could be distinguished from acute cardiogenic pulmonary edema (ACPE) by observing a few differing characteristics [29]. The distribution of ARDS changes are often patchy with one area of the lung more involved than another. The pleural line may be thickened and uneven as a result of inflammatory exudate and effusion often occurs.

ACPE produces bilateral symmetrical B-lines that starts in the lower parts of the lung and progressively involve the upper lung as the severity increases. When fluid floods the alveoli, an air-fluid foam mixture is created, which generates even more B-lines. In severe cases, the multiple B-lines coalesce into a thick white sheet. B-lines in ACPE is quantifiable to determine disease severity and could be used to guide therapy [30, 31]. The commonly found pleural effusion indicates the progressive development of heart failure days to weeks prior to the ACPE episode but cannot be used to determine ACPE severity. Pleural thickening and consolidations are not features of ACPE [29].

#### Pneumonia

LUS is a sensitive tool to pick up changes associated with pneumonia (c.f. CXR). The signs are, however, non-specific and should be interpreted in the context of the clinical findings of a patient.

Most pneumonias begin with interstitial involvement and progress to alveolar infiltration with red cells and serous infiltrates (congestion), followed by deposition of fibrin rich fluid (red hepatisation) and infiltration with inflammatory cells (grey hepatisation). The corresponding LUS signs are B-lines, hypoechoic subpleural defects, and consolidation. Parapneumonic effusions are sometimes present. Fulminant pneumonias may progress to abscess formation.

Acute interstitial pneumonias tend to present with predominantly B-lines in LUS [32], which are unevenly distributed throughout the lungs. Small consolidations and subpleural lesions are common, but there are reports of interstitial pneumonias without these, which is helpful in determining the aetiology [33].

### Acutely dyspneic patient with normal lung ultrasound

Acute asthma and exacerbation of chronic obstructive pulmonary disease (COPD) are reactive airway diseases and do not produce pathophysiologic changes to the lung parenchyma and pleural per se, hence, giving a normal LUS picture. Rarely, in very severe states of air-trapping and lung hyperinflation, pulmonary excursion is severely restricted leading to loss of lung sliding. LUS in asthma and COPD is helpful to exclude other concomitant causes of breathlessness, such as pneumonia and heart failure [34].

### Pulmonary embolism and pulmonary infarction

LUS has shown that pulmonary involvement is common in pulmonary embolism (PE), contrary to common beliefs. PE often produces changes that could be regarded as early infarction [35]. In the initial stage, the alveoli are infiltrated by red blood cells replacing air. Infarctions are typically wedge-shaped subpleural lesions that are homogenously hypoechoic with absent LUS features of air. There may be a linear hyperechoic region within the lesion resembling an air bronchogram. An associated pleural effusion adjacent to the infarction is often seen. An infarction at the lung base gives loss of curtain sign. Over time, the infarcted area will be infiltrated by inflammatory cells and assumes a consolidation appearance. Pulmonary infarctions are most often found in the posterior and lower half of the lung, and LUS has a high sensitivity and diagnostic accuracy for the confirmation of pulmonary infarction [36]. The further workup on these patients require risk-profiling (e.g., Well’s criteria), D-Dimer test, DVT scan, or CT Pulmonary angiogram.

## Conclusions

This review illustrates the fact that many acute lung conditions could be diagnosed readily with LUS. LUS offers us the precision that traditional physical examination of the lung and the chest X-ray could not provide and the ability to make important therapeutic decisions early. Practitioners of LUS should therefore be conversant with the technique and be ready to apply it in primary survey of a dyspneic patient.

## Abbreviations

ACPE: Acute cardiogenic pulmonary edema
ARDS: Acute respiratory distress syndrome
COPD: Chronic obstructive pulmonary disease
CXR: Chest X-rays
LUS: Lung ultrasound
MHz: Megahertz
SLF: Sonographic lung field
